# Levenberg–Marquardt deep neural watermarking for 3D mesh using nearest centroid salient point learning

**DOI:** 10.1038/s41598-024-57360-z

**Published:** 2024-03-23

**Authors:** Modigari Narendra, M. L. Valarmathi, L. Jani Anbarasi, Amir H. Gandomi

**Affiliations:** 1 School of Computer Science and Engineering, Vellore Institute of Technology, Chennai, India; 2 Department of Computer Science and Engineering, Dr. Mahalingam College of Engineering and Technology, Pollachi, India; 3 Faculty of Engineering and IT, University of Technology Sydney, Sydney, NSW 2007 Australia; 4 University Research and Innovation Center (EKIK), Óbuda University, Budapest, 1034 Hungary

**Keywords:** Discrete Gaussian geometric, Salient point detection, Multi-function barycenter, Levenberg–Marquardt, Deep neural network, Computational science, Computer science

## Abstract

Watermarking is one of the crucial techniques in the domain of information security, preventing the exploitation of 3D Mesh models in the era of Internet. In 3D Mesh watermark embedding, moderately perturbing the vertices is commonly required to retain them in certain pre-arranged relationship with their neighboring vertices. This paper proposes a novel watermarking authentication method, called Nearest Centroid Discrete Gaussian and Levenberg–Marquardt (NCDG–LV), for distortion detection and recovery using salient point detection. In this method, the salient points are selected using the Nearest Centroid and Discrete Gaussian Geometric (NC–DGG) salient point detection model. Map segmentation is applied to the 3D Mesh model to segment into distinct sub regions according to the selected salient points. Finally, the watermark is embedded by employing the Multi-function Barycenter into each spatially selected and segmented region. In the extraction process, the embedded 3D Mesh image is extracted from each re-segmented region by means of Levenberg–Marquardt Deep Neural Network Watermark Extraction. In the authentication stage, watermark bits are extracted by analyzing the geometry via Levenberg–Marquardt back-propagation. Based on a performance evaluation, the proposed method exhibits high imperceptibility and tolerance against attacks, such as smoothing, cropping, translation, and rotation. The experimental results further demonstrate that the proposed method is superior in terms of salient point detection time, distortion rate, true positive rate, peak signal to noise ratio, bit error rate, and root mean square error compared to the state-of-the-art methods.

## Introduction

The improvement of computer graphics and multimedia processing has improved the potential use of 3D multimedia content for several applications. As communication networks have evolved to transfer 3D multimedia content over internet more routinely, the security and authenticity of multimedia data have been concerns among the research community. 3D meshes have been used in several domains, such as virtual reality, computer-aided design (CAD) entertainment, and so on. Digital watermarking is a type of information security that embeds cover image or 3D model either in a visible or invisible manner for efficient third-party interference detection. Only an authorized user can then use the extracted information to verify the cover data.

Fragile watermarking authenticates the multimedia data by detecting even the smallest alterations. Robust watermarking embeds a simple image or any kind of data in multimedia content through watermarking to protect the ownership information. The embedding has to be carried out in such a way that the visual distortion of the host model doesn’t affect considerably. The watermarked model must be robust against attacks that can modify the shape of the models indirectly which changes the representation of mesh data without modifying the shape (distortion less attack) or directly (Geometrical attacks) and should be able to retrieve the watermark information with minimum possible loss.

Mesh segmentation of shapes is a well-researched field for watermarking where 3D models with less visual distortion and robust towards attacks are the difficult issues that need to be resolved. The basic ideas behind computer graphics and the animation process are analyzed to deal with the relationship that exists between the model skeleton and the boundaries making segmentation and skeletonization a crucial issue for identifying the elite points to embed the watermark. In contrast to many other models, certain models are consistent in all poses. Any segmentation algorithm must be able to establish consistency across all object families. Common mesh segmentation typically does not directly alter the geometry of the object. Instead, it focuses on locating the boundaries between object parts to segment them. Prior to segmentation, most algorithms set an objective function; if they don’t, they try to find the concavity of the surface models.

3D salient point detection is a vital issue in computer graphics and computer vision. Salient points with local features in 3D models are distinct features crucial for object recognition, pose estimation, motion tracking, and 3D reconstruction and pivotal for efficient data processing and representation. These points are typically characterized by their uniqueness, stability, and importance in representing the overall structure and appearance of the 3D object. The salient points exhibit great stability with respect to geometric transformation and, though to a less extent, to shape deformation.

### Motivation

To achieve optimal stability concerning both rigid and non-rigid transformations, it’s essential to perform an enhanced data embedding process that circumvents visual distortion. Analyzing the intricacy of the 3D mesh model is crucial to pinpoint areas that exhibit visual consistency and semantic importance. This approach ensures that data embedding is facilitated without compromising the visual integrity of the 3D model. Instances of compromised extraction from spatial and frequency domains by potential attackers should be prevented, with a focus on elevating the process through the integration of Artificial Intelligence models.

To attain the high embedding with less distortion a Nearest Centroid Discrete Gaussian and Levenberg Marquardt (NCDG-LV) Deep Learning method for watermark authentication of 3D models is proposed in this paper. More precisely, Salient points were extracted by employing the Nearest Centroid and Discrete Gaussian geometric measure, and 3D models were segmented using map segmentation. In addition, multi-function barycenter is used for embedding and Levenberg Marquardt Deep Neural Network Watermark Extraction is performed to concentrate specifically on the perceptual relevant regions.

### Contribution

The key contributions of the proposed algorithm can be pointed out as,

- The NCDG-LV deep learning method efficiently identifies salient points in 3D models using the Nearest Centroid and Discrete Gaussian geometric (NCDG) approach, leveraging Gaussian curvature to overcome data discretization challenges.
- Map segmentation is achieved through a plane partitioning goal function, minimizing distortion during watermark embedding while segmenting the salient point region.
- Watermark embedding is performed using a multifunction barycenter obtained through nearest centroids, ensuring effective embedding without compromising model integrity.
- The proposed method employs a Levenberg–Marquardt deep neural network for watermark extraction, utilizing the nonlinear characteristics of the optimized kernel to guarantee error stability and weights boundedness.

The remainder of this paper is organized as follows. Section “Literature survey”, discuss various research works performed in the area of 3D model watermarking along with the pros and cons. In Section “Nearest Centroid Discrete Gaussian and Levenberg–Marquardt (NCDG-LV) Deep Learning method”, the proposed Nearest Centroid Discrete Gaussian and Levenberg–Marquardt (NCDG-LV) Deep Learning method is discussed in detail. Experimental results, including comparisons with existing methods, are provided in Section “Results and Discussion”. The conclusion and the future work of this paper are described in Section "Conclusion".

## Literature survey

Zein et al.^1^ performed Fuzzy C-Means (FCM) clustering for watermark insertion through optimized selection of vertices, minimizing perceptual distortion and enhancing robustness against attacks. This work attained Vertex Signal to Noise Ratio SNR values from 122.53 to 140.16 dB and RMSE values between 0.13 * 10^–3^ and 0.27 * 10^–3^ across different models and demonstrated greater resilience to cropping attacks, maintaining high resistance even at 70% cropping level. Liu et al.^2^ leveraged the multiresolution adaptive parameterization of the surface (MAPS) approach to classify vertices into coarse and fine levels for watermark embedding. This selection process strategically embeds watermark information into areas that are less prone to perceptible degradation exhibiting robustness against noise, smoothing, and simplification attacks by correlation values (ρ) ranging from 0.98 to 0.49 for noise attacks, 0.91–0.48 for smoothing attacks, and 0.51–0.56 for simplification attacks across different models and attack parameters.

Hou et al.^3^ performed on layer slicing to overcome watermark removal attacks and employed spread spectrum signal watermarking attaining correlation coefficients of 0.69 for noise attacks, 0.61 for smoothing, 0.565 for quantization, and 0.693 for 5% cropping. Liu et al.^4^ introduced a novel blind watermarking technique for 3D point cloud models where vertices with larger mean curvature are embedded with a secret watermark. With 20% simplification, it achieves 0.4936 accuracy but decreases with increased noise, rotation, and cropping. Borah et al.^5^ proposed a semi-fragile, blind watermarking method called, 3D-Minimum Distortion Angle Quantization Index Modulation (3D-MDAQIM) in spatial domain. The 3D mesh is traversed with a topology-oriented strategy to obtain the elite vertex units for watermark embedding. The watermark embedding is performed by deploying dither modulation to spherical angular values of the identified vertices, causing minimum distortion, but the true positive rate is not focused on understanding the model performance.

Liang et al.^6^ performed Discrete Cosine Transform (DCT) and subsequently encrypted them using the RSA algorithm to embed the watermark. The mapping of float DC coefficients to the integer domain presents challenges, as it necessitates rounding off the float part, potentially resulting in shape loss between the original and recovered 3D models. Peng et al.^7^ enhanced the fidelity of reversible watermarking methods for 3D mesh models by extending 2D region nesting to n-dimensional spaces with the help of a general region nesting technique to embed semi-fragile watermarks based on vertex projection and mesh topology, facilitating authentication and integrity verification of 3D mesh models.

Delmotte et al.^8^ introduced a novel blind watermarking algorithm designed specifically for 3D printed objects that employed subtle modifications to the distribution of surface norms, particularly focusing on the distance between the surface and the center of gravity. Furthermore, the algorithm subdivides the mesh into bins and disperses the data across the entire surface, effectively reducing the impact of local printing artifacts. Peng et al.^9^ performed double modulation to mitigate distortion for transforming a 3D model into the spherical coordinate system through quantization modulation. Watermarks are embedded in both plaintext and encrypted domains and was able to detect malicious tampering across two domains while minimizing distortion with an average distortion of 3.749 × 10^−5^, an average maximum distortion of 0.999 × 10^−4^, an average signal-to-noise ratio (SNR) of 90.870 across 7 models and zero Bit Error Rate.

Bhardwaj et al.^10^ performed a novel reversible data hiding technique for 3D mesh models in the compressed domain while preserving the original mesh topology and vertex order, facilitating accurate message extraction and seamless reconstruction of the cover 3D mesh model attaining a PSNR value of 96.40 dB for an embedding rate of 6.94 bits per vertex with Hausdorff distance of 0.2358. Peng et al.^11^ proposed a method using virtual polygon projection where extraction is based on vertex positions overcomming tampering attack. With double modulation strategy, the average and maximum distortion are decreased by 0.0411 and 0.1608, and the average SNR is increased by 2.5649 compared with IQIM (Fei Peng et al.^12^), respectively.

Lee et al.^13^ performed zero-watermarking method that includes coordinate correction, spatial partition, gene feature extraction, and genotype detection. The statistical examination of distortion attacks on a zero-watermarking method demonstrated robust resistance to noise addition (1.00 ratio), cropping (correlation > 0.88), and subdivision attacks (correlation > 0.94 in the midpoint scheme). Peng et al.^14^ performed spherical crown volume division to minimize embedding distortion and topological transformations during watermark generation. By grouping the converted spherical coordinates based on their one-ring neighborhood, tampering localization accuracy is improved. Yang et al.^15^ analyzed a steganalysis algorithm to enhance the 3D watermarking techniques developed by Cho et al. for detecting the embedded watermark through bimodal distribution of histogram bins’ means/variances based on radial coordinates. Rather than integrating each watermark bit within a continuous statistical feature this model embedded within a discrete statistical measure, particularly focusing on the variance between two adjacent bins. The steganalysis algorithm achieves 98.65% accuracy in estimating the number of bins in the variance-based method and demonstrates robustness against noise addition, smoothing, quantization, subdivision, and simplification, maintaining high correlation coefficients even after significant attacks.

Jiang et al.^16^ performed bit-stream encryption to embed the watermark using data-hiding key in least-significant bits. Leveraging spatial correlation within natural mesh models, ensured the good recovery of the original mesh achieving an embedding rate of 0.7 bits per vector. On the Princeton Shape Retrieval dataset, the average error rate stands at 4.2%, while with the Stanford 3D Scanning Repository, error rates range between 9.7 and 11.4%. Nassima et al.^17^ derived salient points using a 3D salient point detector based on the Auto Diffusion Function, followed by segmentation of the 3D model into regions anchored to these salient points. The watermark is then inserted into each region using the embedding technique of Cho et al. By employing geodesic Voronoi segmentation, the surface is divided into cells associated with feature points, allowing for precise watermark embedding and extraction and able to achieve Haussdorff distance (HD) values ranging from 0.33 to 10.7 × 10^–3^ and minimal roughness.

Niu et al.^18^ discusses the use of Laplace–Beltrami eigen functions that are invariant to rigid transformations to extract salient points representing distinctive regions computed based on specific criteria, including clustering and geodesic distance computations. Feng et al.^19^ presented a novel mesh visual quality metric that integrated saliency considerations to estimate local distortions in the mesh. Li et al.^20^ performed multiresolution 3D wavelet analysis, Laplacian smoothing and normalization and Wavelet Coefficient for watermarks embedding and extraction. Zhang et al.^21^ enhanced Reversible Data Hiding approach using prediction-error expansion and embedded the watermark in adjacent neighbors generating a ring pattern for easy prediction vertex. Data bits are embedded reversibly into 3D mesh models via operations like expansion, shifting, and LSB replacement with smoothness sorting and a twice-layered strategy and achieved a good SNR of 45 dB with 0.7 bits per vertex embedded.

The need for robust and secure methods to protect 3D models has led to an increase in the significance of research in the area of deep learning approaches and 3D mesh watermarking in recent years. Deep learning methods promise great embedding capacity, robustness against attacks, and imperceptibility of embedded watermarks, providing distinct advantages in capturing intricate features and learning complicated mappings within 3D meshes. Several obstacles must be overcome by researchers as they work in this field, including a lack of labeled training data, overfitting, interpretability issues, adversarial attacks, and computational complexity. Notwithstanding these obstacles, there is a lot of potential for revolutionizing digital material security and authentication through the investigation of deep learning techniques for 3D mesh watermarking.

Zhu et al.^22^ employs a Graph Attention Network (GAT) to extract local features from vertex relations, providing robustness even after mesh simplification. Additionally, an attack layer perturbs the watermarked vertices to augment robustness against cropping, noise, rotation, translation, and scaling attacks. Wang et al.^23^ introduces the deep 3D mesh watermarking network, where the curvature consistency loss function is created to limit the local geometry smoothness of watermarked meshes in order to maintain the visual quality of 3D meshes. The architecture includes embedding, extracting sub-networks, and attack layers, employing topology-agnostic graph convolutions for flexible mesh handling. The approach tried to ensure robustness with adaptive attack layers and maintains visual quality via a curvature consistency loss for smooth watermarked mesh geometry.

Abouelaziz et al.^24^ computed visual saliency using a method based on mean curvature and Gaussian filtering to select relevant patches from rendered 2D projections of the 3D model. Then a simple local contrast normalization is applied to address illumination and contrast variations. For feature learning and quality score estimation, three pre-trained CNN models (AlexNet, VGG, and ResNet) are fine-tuned, and their extracted features are combined using Compact Multi-linear Pooling (CMP) to interact multiplicatively. The combined features are fed into fully connected layers followed by a regression layer for quality score prediction. This approach showcases the integration of deep learning and saliency analysis for efficient and accurate quality assessment of 3D meshes.

The advancements in 3D mesh watermarking techniques have seen significant progress, with various methods addressing different aspects of robustness, imperceptibility, and resilience against attacks. From employing clustering algorithms like Fuzzy C-Means for vertex selection to leveraging multiresolution adaptive parameterization and spread spectrum signals for watermark embedding, researchers have explored diverse approaches to enhance the security and authentication of 3D models. Techniques such as deep learning-based approaches, reversible data hiding, and graph attention networks have shown promise in overcoming challenges like overfitting, interpretability issues, and adversarial attacks. Despite obstacles such as a lack of labeled data and computational complexity, the field of 3D mesh watermarking is poised for further development, especially with the integration of deep learning methodologies, saliency analysis, and robust watermark embedding strategies. These advancements hold the potential to revolutionize digital material security and authentication, paving the way for more secure and reliable methods in the realm of 3D model protection.

## Nearest Centroid Discrete Gaussian and Levenberg–Marquardt (NCDG-LV) Deep Learning method

**Figure 1:**
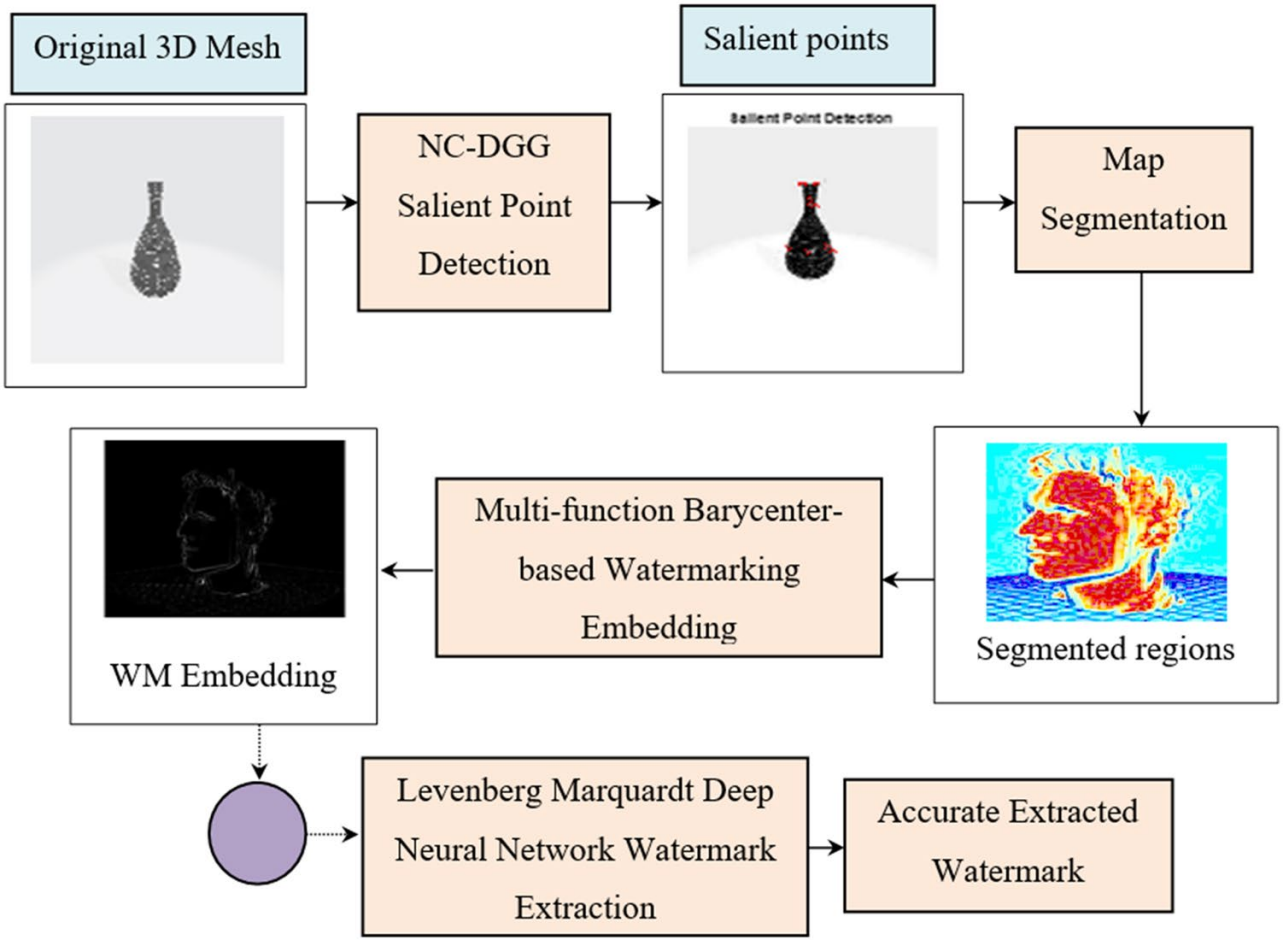
Nearest Centroid Discrete Gaussian and Levenberg–Marquardt Deep Learning method.

Structure of the proposed method for 3D mesh authentication is demonstrated in Fig. 1. Initially, the Nearest Centroid and Discrete Gaussian geometric (NC–DGG) Salient Point Detection model is used to detect the optimally and computationally efficient salient points. Second, with the detected salient points, 3D Mesh model is segmented into regions using map segmentation. Third, the watermark is inserted into each region using the multi-function barycenter-based Watermarking Embedding model. Finally, the watermark is extracted by employing the Levenberg–Marquardt deep neural network watermark extraction model for achieving good imperceptibility and robustness against attacks.

### 3D mesh model representation

**Figure 2:**
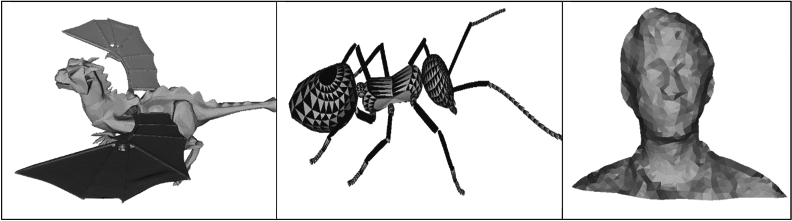
Some of the 3d mesh models used in the proposed method.

A 3D mesh has structural construct of a 3D model consisting of polygons. Triangle mesh has type of polygon mesh. It includes a set of triangles in three dimensions that are linked with edges or vertices. 3D mesh model has denoted as three-dimensional object that includes points (i.e., vertices), lines (i.e., edges), and faces (i.e., surfaces). These elements are employed to refer the shape of the modeled 3D object. The 3D mesh model is comprised of a set of vertices ‘$V$’ in Cartesian coordinates and a set of edges ‘$E$’ represented as ‘$G=\left(V,E\right)$’. Let us consider that ‘${V}_{i}$’ corresponds to the vertex indexed by ‘$i$’ and is designated by its corresponding 3D coordinates ‘$\left({P}_{i}, {Q}_{i}, {R}_{i}\right)$’. The vertices group that is adjacent to a neighborhood vertex ‘${V}_{i}$’ is referred to as ‘$1-ring$’ of the vertex, and the number of vertices that is adjacent to neighborhood vertex ‘${V}_{i}$’ in the ‘$1-ring$’is referred to as the degree of the vertex ‘${V}_{i}$’. In a similar manner, the ‘$k-th ring$’ neighborhood vertices around vertex ‘${V}_{i}$’ can be obtained by means of the K-Nearest Centroid Discrete Gaussian geometric measure. Some of the 3D mesh models used in the proposed work is shown in Fig. 2.

### Nearest centroid & discrete Gaussian geometric (NC–DGG) salient point detection

The salient characteristics of 3D mesh models are distinctiveness, resilience, invariance, repetition, localization, semantic meaning, efficiency, and scalability. These geometric properties are essential and has significant importance in several applications, such as shape analysis, recognition, and embedding. These points typically include regions with significant curvature, points of utmost magnitude, angular points, and locations along the boundary. Identifying them is crucial for acquiring complex features, essential structural elements, and unique geometric properties to embed secrets in the mesh model. Embedding approaches, utilizing salient points, can efficiently encode and depict geometric information while exhibiting resilience against noise, distortion, and geometric attacks.

**Figure 3:**
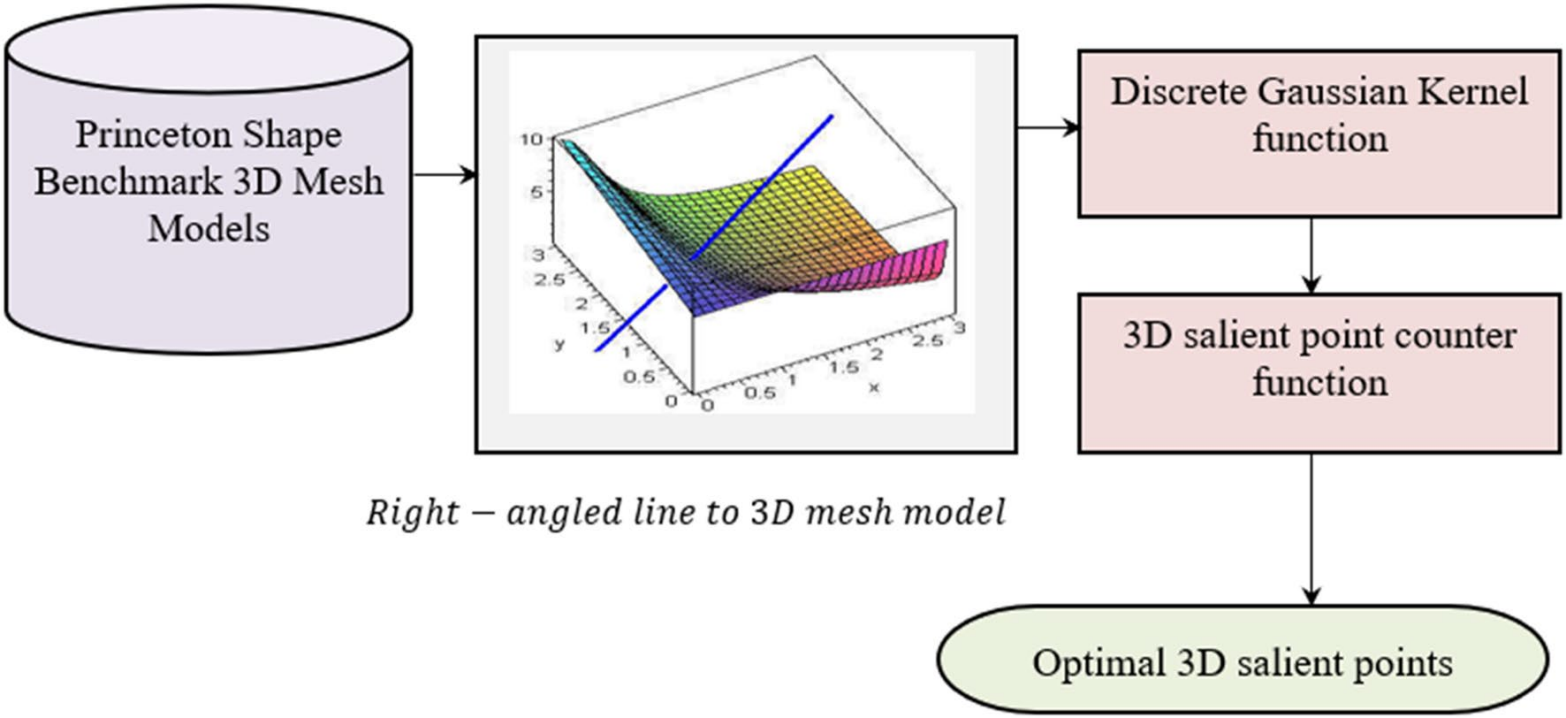
Structure of Nearest Centroid and Discrete Gaussian geometric (NC–DGG) Salient Point Detection model.

Figure 3 shows the structure of the Nearest Centroid and Discrete Gaussian geometric (NC–DGG) salient point detection model. The 3D mesh model salient point detection method, is analyzed using the 3D models obtained from Princeton Shape Benchmark to identify prominent features from the geometric attributes and its spatial relationships. Initially, the method employed the nearest centroid technique to identify central points within the mesh, serving as potential candidates for salient points. Leveraging an Optimal 3D salient point detection function, the method assesses various geometric properties and vertex densities to discern salient features from background elements. By applying a Discrete Gaussian Kernel function, local distributions of vertex densities are computed, illuminating regions of higher significance. Subsequently, a 3D salient point counter function quantifies the saliency of each candidate point based on its proximity to dense vertex clusters and its contribution to shape distinctiveness. The evaluation extends to analyzing the Euclidean distance of neighborhood vertices, which aids in discerning salient features amid the mesh’s structural complexity. By scrutinizing the distribution and spatial relationships of salient points, including the alignment of right-angled lines connecting neighboring vertices, the method systematically identifies and characterizes salient features within the 3D mesh model, enabling effective feature extraction and shape analysis for diverse computational applications.

As shown in Fig. 3, for every vertex $v$ on a 3D mesh model, $n$ denotes the normalized vector. Only one right-angled plane exists for this vertex $v$, which is estimated as:

1 $${n}^{T}{\left[p-{p}_{v}, q-{q}_{v}, r-{r}_{v}\right]}^{T}=0$$

where (p, q, r) denotes the coordinates of vertex ‘$v$’ and $\left({p}_{v}, {q}_{v}, {r}_{v}\right)$ represents the vertex normal. The average distance of the kth ring neighborhood vertices around vertex ‘${V}_{i}$’ is formulated as:

2 $${Dis}_{ij}=\frac{{n}^{T}{\left[{p}_{ij}, {q}_{ij}, {r}_{ij}\right]}^{T}-{n}^{T}{\left[{p}_{v}, {q}_{v}, {r}_{v}\right]}^{T}}{N}$$

where ‘$\left({p}_{ij}, {q}_{ij}, {r}_{ij}\right)$’ corresponds to the ‘$j-th$’ coordinate of the 3D mesh model in ‘${V}_{i}\left(k\right)$’ for ‘$N$’ different samples. Consider that ‘$M\left(p,q,r\right)$’ denotes the 3D mesh model acquired from ^25^. New 3D mesh models ${M}_{\alpha }\left(p,q,r\right)$ are generated around vertices in the original mesh model. These new models are created based on the neighborhood vertices surrounding the target vertex and are related to the original mesh through the application of a discrete Gaussian kernel function. The purpose of employing this function is to determine the salient scale, providing insight into the significant geometric features within the local neighborhood of the target vertex $v$.

3 $${M}_{\alpha }\left(p,q,r\right)=M\left(p,q,r\right)*DG\left(p,q,r,\alpha \right)$$

4 $$T\left(n,\alpha \right)={e}^{-\alpha }{M}_{i}\left(\alpha \right)$$

5 $$DG\left(p,q,r,\alpha \right)= \sum_{n=-\infty }^{\infty }f\left(p-n\right)f\left(q-n\right)f\left(r-n\right) T\left(n,\alpha \right)$$

where ‘$\alpha =\left(\varepsilon , 2\varepsilon , 3\varepsilon ,\dots .n\varepsilon \right)$’ corresponds to the standard deviation of the respective 3D Discrete Gaussian Kernel filter ‘$DG$’ and ‘$\varepsilon$’ refers to the distance of the main slant in the nearest neighbor vertex of the model. Based on the distance geometric measure, a 3D salient point counter function is defined that authorizes us to extract non-cognitively significant salient points from 3D mesh models. For any vertex ‘${V}_{i}$’ in a 3D mesh model, we utilize the counter function as follows:

6 $$\beta = \frac{{Dis}_{s}^{\prime}-Min \left({Dis}_{s}^{\prime}\right)}{Max \left({Dis}_{s}^{\prime}\right)-Min \left({Dis}_{s}^{\prime}\right)}, where\, {Dis}^{\prime}=\sum_{i=1}^{n}{Dis}_{i}^{\prime}$$

where ‘$\beta$’ corresponds to the distance geometric measure of vertex ‘$v$’ in scale ‘$s=\mathrm{1,2},3,\dots n$’, which is modeled based on the minimum distance function ‘$Min \left({Dis}_{s}^{\prime}\right)$’ and maximum distance function ‘$Max \left({Dis}_{s}^{\prime}\right)$’ in which ‘${Dis}^{\prime}$’ represents the summation. With the obtained 3D salient point counter function for every vertex ‘$v$’, the value of the counter function is compared for every vertex ‘$v$’ in its Nearest Centroid rings. If the value of ‘$\beta$’ is greater than all the values ‘$\beta$’ in its Nearest Centroid rings, the vertex ‘$v$’ is said to be the selected optimal salient point; otherwise, the vertex ‘$v$’ is not a salient point. The pseudo code representation of Nearest Centroid and Discrete Gaussian salient point detection is given below.

**Algorithm 1:**
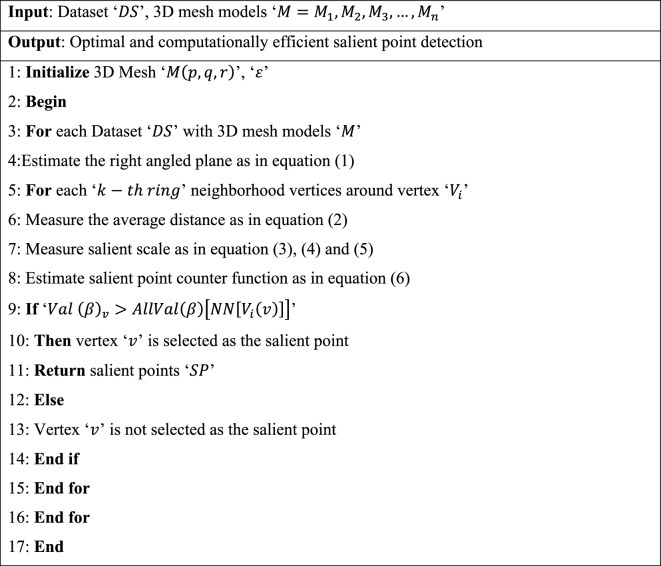
Nearest Centroid and Discrete Gaussian.

### Map segmentation

After acquiring the salient points, the 3D mesh models are further segmented into distinct sub regions Green plane, and Blue plane with the plane partitioning map function. The map functions are separated geographic regions in the model, associated to the salient points, and constructed by means of a plane partitioning goal function. The planes are then divided into ‘$n$’ sub regions $SR={SR}_{1}, {SR}_{2}, {SR}_{3}, \dots .,{SR}_{n}$ in such a manner that each region consists of approximately a ratio of ‘$\frac{1}{n}$’ green partitions and ‘$\frac{1}{n}$’ blue partitions. For each subregion SR_i_, Eq. (7) computes the sum of the intersections of the green plane ‘G’ (Plane_G_) and the blue plane ‘B’ (Plane_R_). The maximum value across these intersections represents the evolved regions resulting from the segmentation process.

7 $$G\left({SR}_{1}, {SR}_{2}, {SR}_{3}, \dots .,{SR}_{n}\right)=MAX \left(\left[\frac{{Plane}_{G}\cap {SR}_{i}}{n}\right]+\left[\frac{{plane}_{B}\cap {SR}_{i}}{n}\right]\right)$$

### Multi-function barycenter and Levenberg Marquardt Deep Learning Model

**Figure 4:**
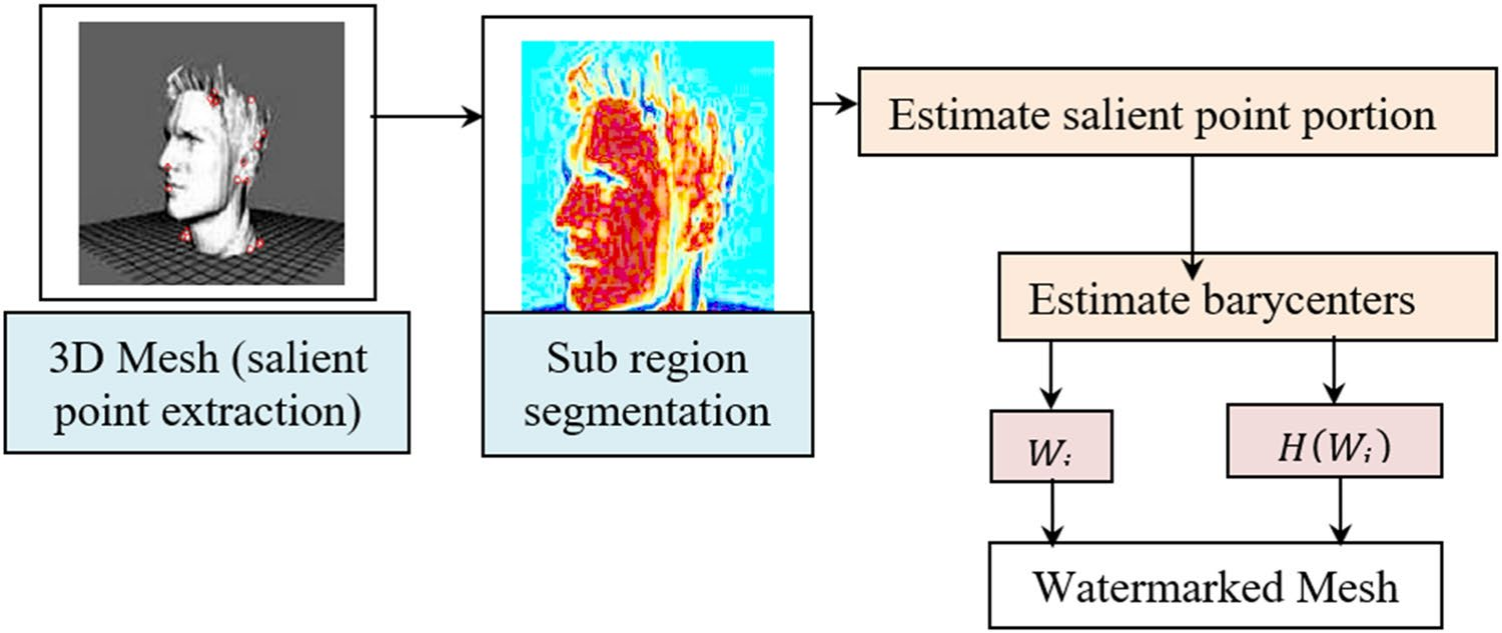
Structure of Multi-function Barycenter-based Watermarking Embedding Model.

Following the segmentation process, the multi-function barycenter is utilized to perform watermarking embedding. Figure 4 shows the structure of the multi-function barycenter-based watermarking embedding model.

The watermark ‘$W=\left({W}_{1}, {W}_{2}, {W}_{3}, \dots ,{W}_{n}\right)$’ is embedded by persuading each ‘${W}_{i}$’ with a small shift in a subset of ‘$V$’. A vertex $v$ is noted as ‘$V\left({p}_{v},{q}_{v},{r}_{v}\right)$’ or ‘$V\left({p}_{v}{\prime}, {q}_{v}{\prime}, {r}_{v}{\prime}\right)$’ before or after embedding, respectively. Calculate displacement factor $l$ used to adjust the positions of the barycenters within the mesh as shown in Eq. (8). Higher values of $l$ could lead to larger displacements, resulting in more significant changes in the mesh geometry. For each being a salient point ‘$v$’, its ‘${p}_{v}$’ value is divided by the parameter ‘${G}^{w}$’ that controls granularity or scale of the embedding to nearest integer. By controlling the scale of the adjustments made to ‘${p}_{v}$’ as shown in Eq. (8), the watermark can be embedded in a way that is resilient to common attacks or transformations applied to the mesh model, such as scaling, rotation, or translation, ensures consistency across the embedding process, allowing for reproducible results and predictable behavior when embedding the watermark into different regions of the mesh or across multiple meshes.

8 $$l=\left[\frac{{p}_{v}}{{G}^{w}}\right]; {p}_{v}=\left[l*{G}^{w}\right]$$

Next, for each salient point its ‘${q}_{v}$’ and ‘${r}_{v}$’ barycenters are measured by obtaining the mean of the coordinates of its Nearest Centroids as:

9 $${q}_{v}^{c}=\frac{1}{\left|NC\left(v\right)\right|}\sum_{NC\left(v\right)}{q}_{v}$$

10 $${r}_{v}^{c}=\frac{1}{\left|NC\left(v\right)\right|}\sum_{NC\left(v\right)}{r}_{v}$$

11 $${M}_{v}^{E}=\left[{p}_{v}+{q}_{v}^{c}+{r}_{v}^{c}\right]+{Sh}_{j}$$

where ‘$NC\left(v\right)$’ refers to the set of ‘$v{\prime}s$’ nearest centroid value; and ‘$\left|NC\left(v\right)\right|$’ corresponds to the size of ‘$NC\left(v\right)$’. Finally, the watermark ‘${W}_{i}$’ and hash value ‘$H\left({W}_{i}\right)$’ are embedded in ‘${M}_{v}^{E}$’formulated as given below:

12 $$S={M}_{v}^{E}+{W}_{i}+H\left({W}_{i}\right)$$

Finally, $S$ denotes the embedded watermark, Cryptography hash function is used for calculating $H\left({W}_{i}\right)$, and the length of $H\left({W}_{i}\right)$ is 128 bit. The embedding perturbs ‘${M}_{v}^{E}$’ toward the original value ‘$\left({p}_{v}, {q}_{v}, {r}_{v}\right)$’ with a small shift ‘${Sh}_{j}$’, which is always less than ‘$j=\left(\mathrm{2,3}\right)$’.

### Levenberg–Marquardt deep neural network watermark extraction

**Figure 5:**
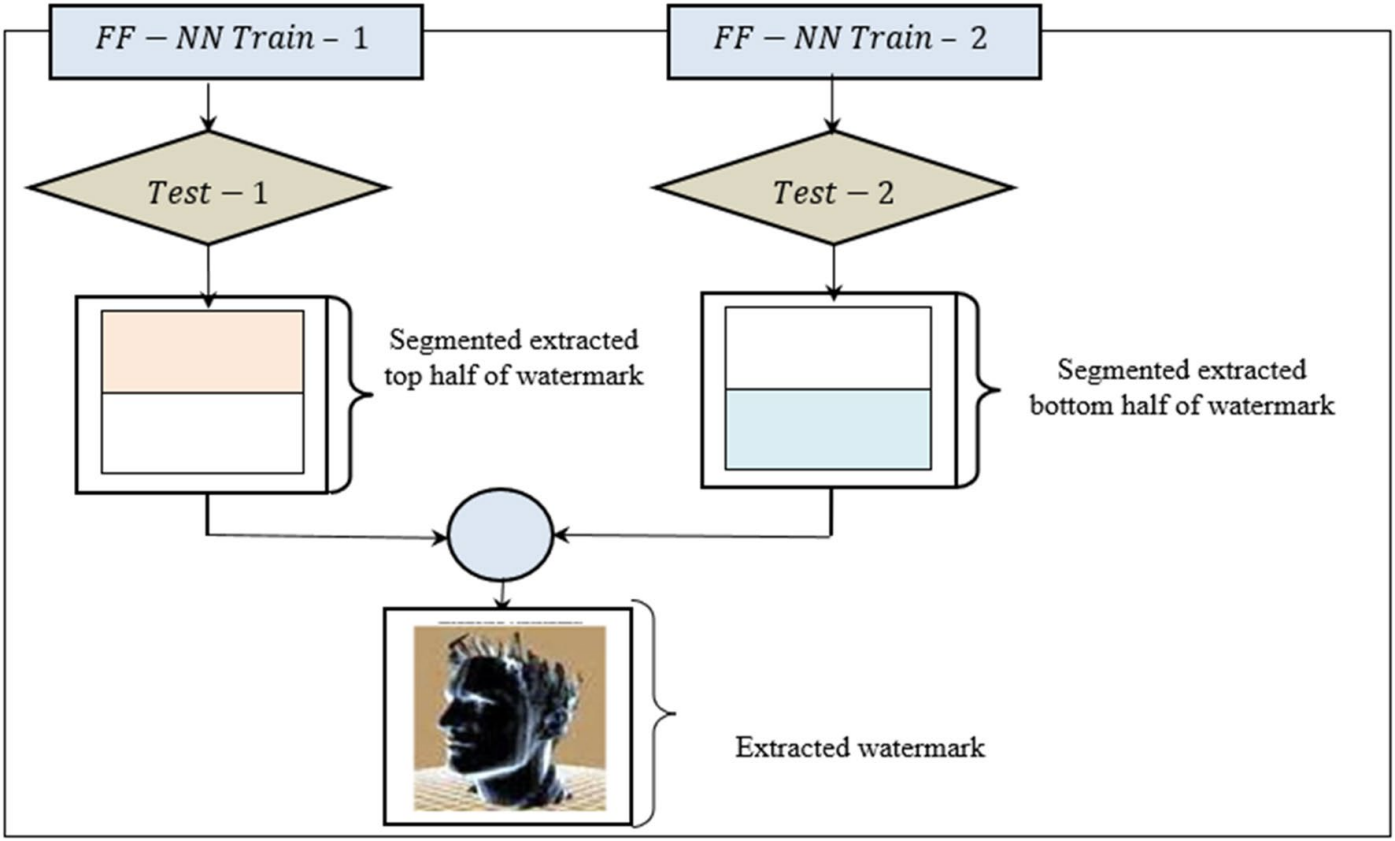
Levenberg–Marquardt deep neural network Watermark Extraction Model.

In the watermark extraction stage, ‘${G}^{w}$’ and ‘$H$’ serve as the pivotal elements for detecting any malicious patterns. In the proposed work, with the objective of improving the precision and recall involved in the watermark embedding and extraction process, a Levenberg**–**Marquardt deep neural network watermark extraction model is used. Figure 5 displays the structure of Levenberg**–**Marquardt deep neural network watermark extraction model.

**Figure 6:**
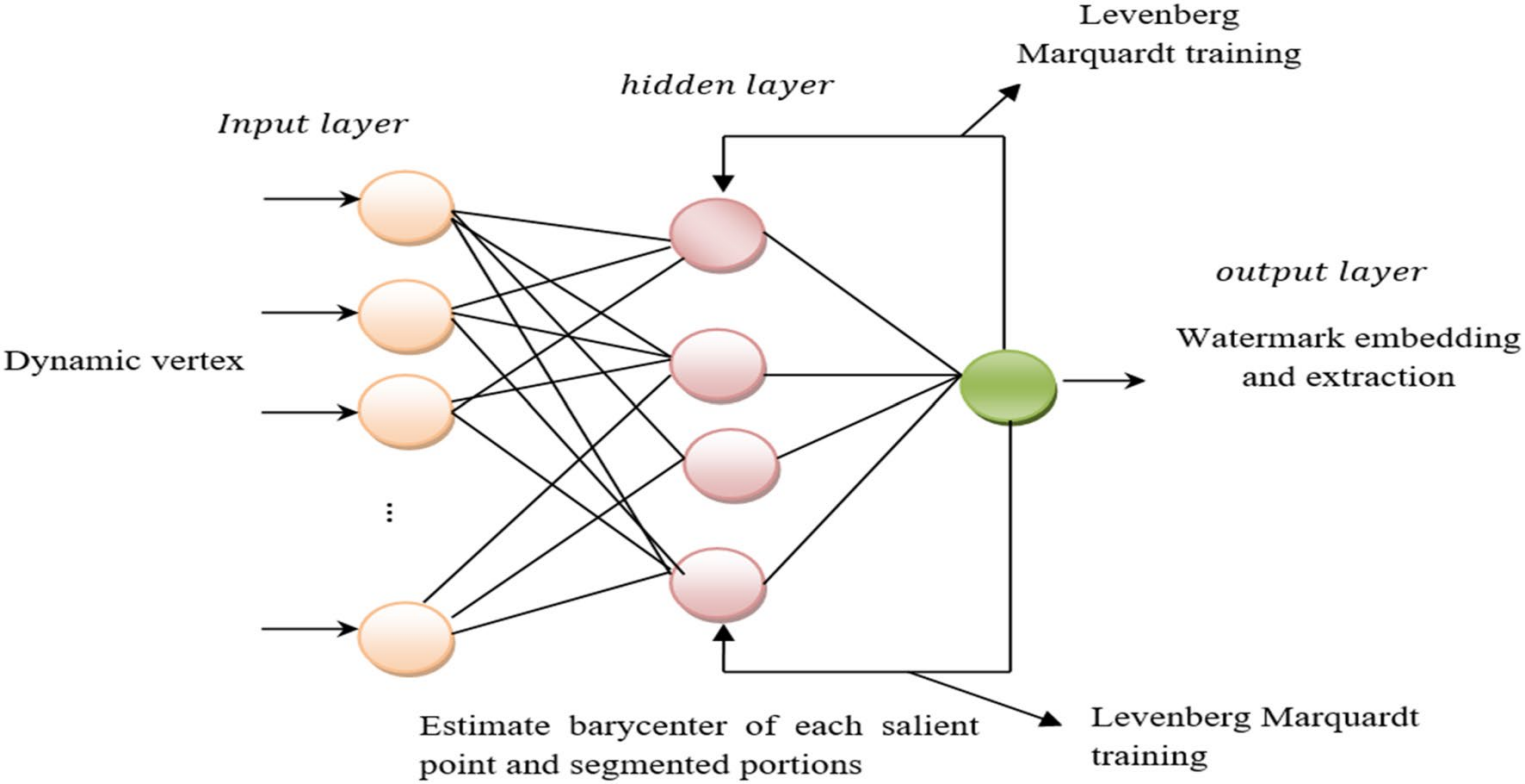
Structure of Levenberg–Marquardt deep neural network.

Figure 6 demonstrates the structure of Levenberg**–**Marquardt deep neural network to perform watermark extraction. It includes an input layer, hidden layer, and output layer. In the input layer, ‘${V}_{i}$’ is a dynamic vertex that passes over the entire 3D Mesh model, initiating from ‘${V}_{i}$’, to inspect the model. In the hidden layer, the corresponding ‘${q}_{v}$’ and ‘${r}_{v}$’ barycenters of each salient point and segmented portions are estimated using Eqs. (9) and (10), respectively, and then used to extract the watermarks as follows:

13 $${H}^{\prime}=\left({p}_{v}^{c}-{p}_{v}^{\prime}\right) \cup \left({q}_{v}^{c}-{q}_{v}^{\prime}\right) \cup \left({r}_{v}^{c}-{r}_{v}^{\prime}\right) \cup$$

For each vertex ‘${V}_{i}$’, the weights associating the input-hidden and hidden-output layers are updated according to the desired output, and the process is iterated until the convergence. Subsequently, the network trained model is utilized for classification of the test set. This process is performed two times, one for the top half segmented regions ‘$TH$’ and the second for the bottom half ‘$BH$’ segmented regions. This is formulated in Eqs. (14) and (15):

14 $$f=\left\{{Train}_{i,j}, {Label}_{i,j}\right\}=\left[\left({F}_{i,j}\left(1\right)\right), \left({F}_{i,j}\left(2\right)\right),{W}_{i,j}\right] where\, {Label}_{i,j} \in TH, B$$

15 $${W}{\prime}=f \left({Test}_{i,j}\right)=\left\{\left({F}_{i,j}\left(1\right)\right), \left({F}_{i,j}\left(2\right)\right)\right\}$$

Finally, the output layer constitutes the extraction result, i.e., distorted regions or non-distorted regions. To speed up the watermark extraction process and minimize the memory, the Levenberg– Marquardt back-propagation is employed while training network by setting the learning rate to 0.01. The pseudo code representation of multi-function barycenter and Levenberg–Marquardt Deep Learning Extraction is given below.

**Algorithm 2:**
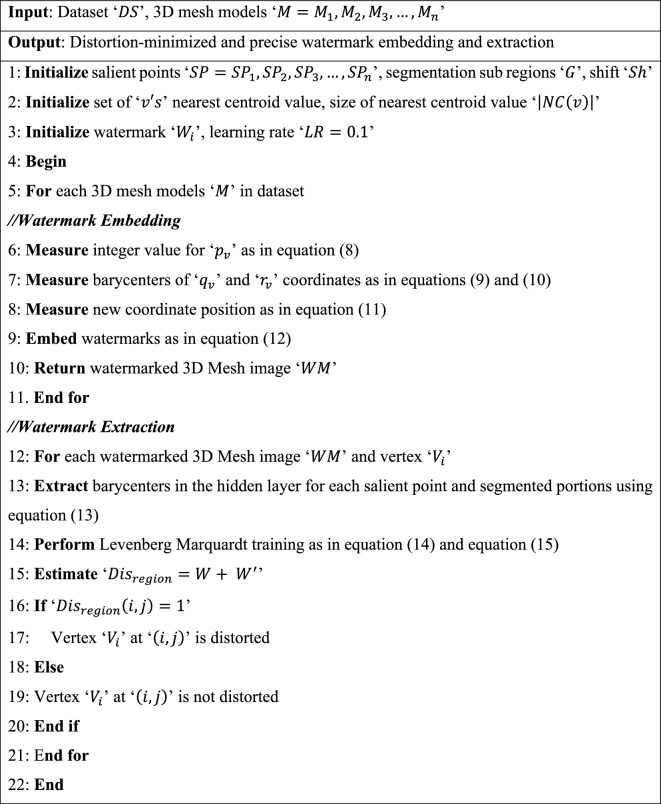
Multi-function Barycenter and Levenberg–Marquardt Deep Learning Extraction.

## Results and discussion

The proposed Centroid Discrete Gaussian and Levenberg–Marquardt (NCDG-LV) model for embedding and extraction of watermark in 3D models are implemented in MATLAB R2023a. The experiments were conducted in a PC with the hardware specification of Windows 10, core i7 3.40GHZ Processor, 16 GB RAM, 1 TB (Solid state drive) and Graphics Card NVIDIA Quadro RTX 5000 16 GB.

### Dataset

The proposed Centroid Discrete Gaussian and Levenberg–Marquardt (NCDG-LV) was tested on the Princeton Shape Benchmark dataset^25^. The benchmark dataset comprises of a 3D polygonal models obtained from the World Wide Web. For each 3D model, there is polygonal geometry model and a JPEG image file which is a thumbnail view of the model. The dataset includes 1814 models of various sizes and mesh pattern. It is useful to estimate shape-based retrieval and analysis, involving progress in matching, classification, clustering, and recognition of 3D models. Herein, the benchmark dataset was divided into a training and test set. The training et consists of 1451 models, and the test database consists of 363 models.

### Estimation of salient point detection time

The detection of salient point is an essential parameter since it reveals the portions to be watermarked in a precise manner during watermark embedding. The salient point detection time is measured as follows:

16 $${SPD}_{t}=\sum_{i=1}^{n}{M}_{i}*Time \left[\beta \right]$$

**Figure 7:**
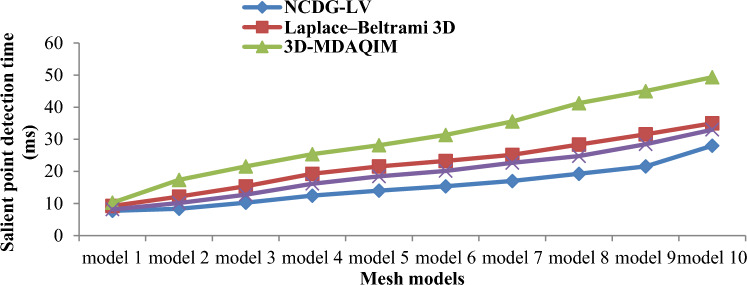
Salient point detection time of first 10 models.

The salient point detection time ‘${SPD}_{t}$’ is measured in milliseconds (ms) for each 3D mesh models used in the watermarking process ‘${M}_{i}$’ and the time consumed in detecting the actual salient point ‘$Time \left[\beta \right]$’. Herein, salient point detection is performed on various 3D mesh models, and the resultant results are shown in Fig. 7. The estimation was performed for the models with size lower to higher showing the time taken for small size models are less and inverse for the other case.

Due to the different sizes of 3D mesh models, the salient point detection time varied. The salient point detection time complexity is significantly smaller for a single 3D mesh model and higher for large number of models. For the first simulation run, the salient point detection time obtained by the proposed NCDG-LV method, Laplace–Beltrami 3D^18^, 3D-MDAQIM^5^, and Deep 3D mesh watermarking network^23^ was determined to be 7.75, 9.25, 10.25 and 8.15, respectively. The better salient point detection time of NCDG-LV, which was 30%, 48% and 18% faster than the three other respective methods, can be attributed to the Nearest Centroid function employed via the Discrete Gaussian Kernel function.

### Embedding and extraction

**Figure 8:**
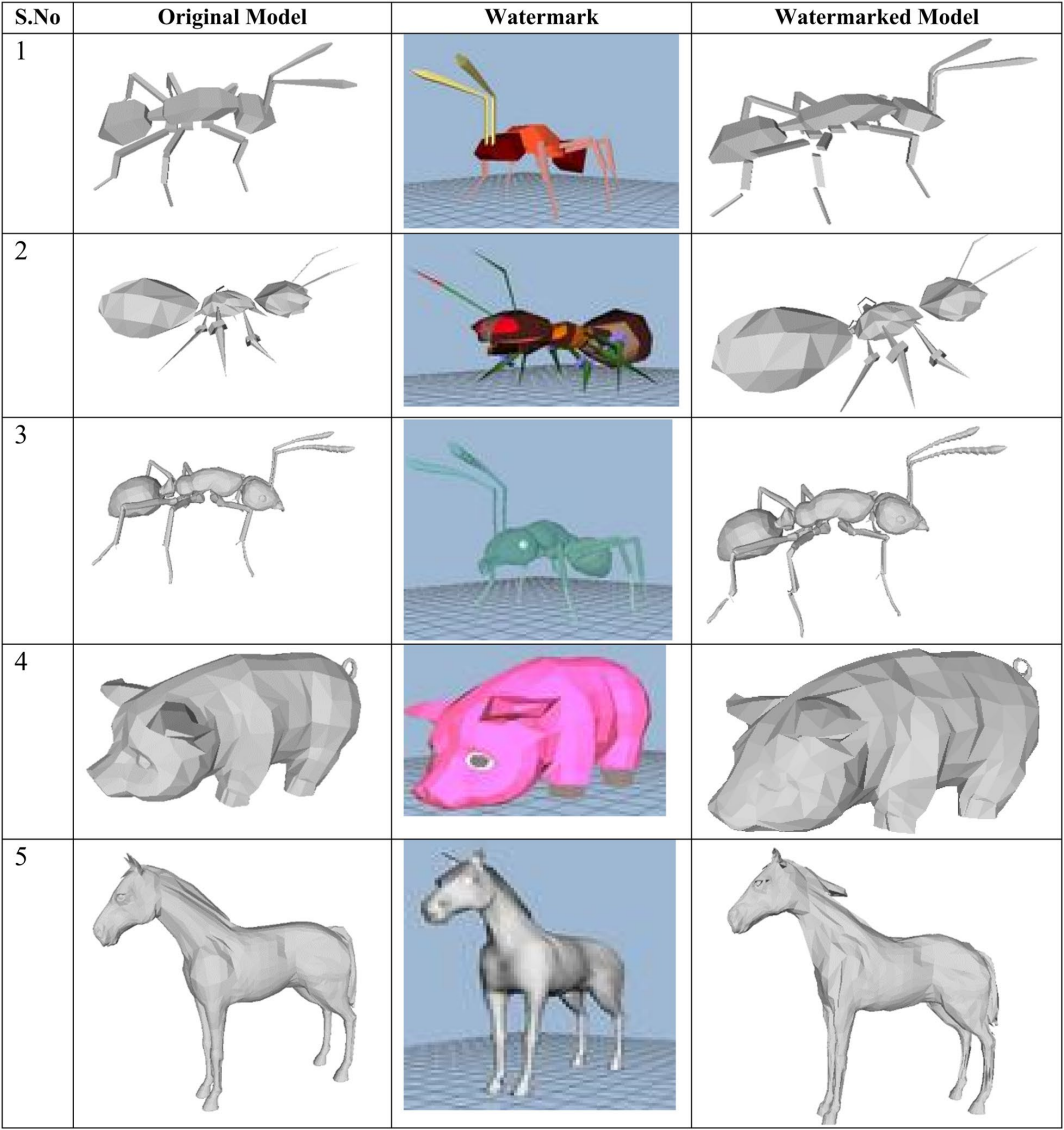
Watermark embedding on 3D mesh models.

Watermarks are embedded into the segmented regions obtained by plane partitioning mapping through the extracted salient points. The watermark which is the thumbnail view of the 3D model is embedded into the resulting region through multi-function barycenter and is shown in Fig. 8. The resulted 3D model after embedding shows some visible visual distortions depending on the size of the original 3D model and the watermark. The watermarked model is trained and tested to extract the watermarks from the salient point through Levenberg–Marquardt deep neural network.

### Performance evaluation of centroid discrete Gaussian and Levenberg–Marquardt

To evaluate the performance of the proposed method, four objective parameters, including watermark extraction against different attacks, spatial detection time, distortion rate, true positive rate, and PSNR are considered.

#### Peak Signal to Noise Ratio

The peak signal-to-noise ratio (PSNR) is evaluated to evaluate the imperceptibility based on the mean square error using the Eq. (17) and the results obtained from different methods are shown in Table 1:

17 $$PSNR=10*{log}_{10}\left(\frac{Max}{MSE}\right)$$

where MAX is the maximum possible value coordinate.

**Table 1:** PSNR of NCDG-LV method, Laplace–Beltrami 3D^18^, and 3D-MDAQIM^5^, Deep 3D mesh^23^ on a set of 10 distinct 3D mesh model.

Model	NCDG-LV	Laplace–Beltrami 3D	3D-MDAQIM	Deep 3D mesh
Model1	56.08	54.15	51.22	55.48
Model2	58.58	56.08	52.56	57.56
Model3	52.56	46.54	45.20	50.02
Model4	58.58	54.15	51.22	56.43
Model5	54.15	52.56	50.06	53.56
Model6	56.08	54.15	51.22	55.11
Model7	51.22	49.04	47.30	50.02
Model8	56.08	54.15	52.56	55.22
Model9	52.56	51.22	50.06	51.87
Model10	54.13	53.12	52.18	48.23

Root Mean square Error (RMSE) given in Eq. (18) identifies the geometrical distortion between two meshes, where $v, \mathop v\limits^{\prime }$ refers to the vertices of original mesh M and deformed meshes surface ${{\text{M}}}^{\mathrm{^{\prime}}}$, and N refers to number of vertices in the mesh model.

18 $${\text{d}}_{{{\text{rmse}}}} \left( {{\text{M}},{\text{M}}^{\prime } } \right) = \sqrt {\frac{1}{N}\mathop \sum \limits_{i = 1}^{N} \left( {v - \mathop v\limits^{\prime } } \right)^{2} }$$

Table 1 provides a comparative analysis of the PSNR of the four different methods. For fair comparison, the above analysis was conducted using 10 3D mesh models, namely rabbit, vase, bee, face, horse, table, bird, spider, ant, and dog models. In PSNR with attacks, 14.7 KB image size is considered to evaluate the experiments. The average PSNR of proposed NCDG-LV for 10 models is 55.02, whereas the PSNR of existing^5,18,23^ is 52.52, and 50.38, and 53.35 respectively.

#### Bit Error Rate (BER)

Bit error rate measures the accuracy of watermark extraction, representing the ratio of incorrectly decoded bits to the total number of bits in the watermark as given in Eq. (19). Achieving a lower bit error rate with better embedding capacity plays a major role in watermarking scheme and the results obtained from different methods are shown in Table 2:

19 $$BER\left(M,{M}^{\prime}\right)=1-\frac{1}{{n}_{b}}\sum_{n=1}^{{n}_{b}}\delta \left({m}_{i},{m}_{i}^{\prime}\right)$$

where ${n}_{b}$ is the number of bits embedded, and $\delta$ is the Kronecker delta function.

**Table 2:** Bit error rate of NCDG-LV method, Laplace–Beltrami 3D^18^, and 3D-MDAQIM^5^, Deep 3D mesh^23^ on a set of 10 distinct 3D mesh model.

Model	NCDG-LV	Laplace–Beltrami 3D	3D-MDAQIM	Deep 3D mesh
Model1	2.15	3.25	4.00	2.85
Model2	2.35	3.85	4.15	3.15
Model3	3.15	3.95	4.35	3.65
Model4	3.85	4.15	4.85	4.05
Model5	4.15	5.00	5.15	4.65
Model6	5.35	5.65	5.25	5.45
Model7	6.25	7.00	8.15	6.85
Model8	8.00	7.35	8.35	7.75
Model9	9.15	8.00	9.25	8.45
Model10	12.00	8.25	12.55	11
Average	5.64	5.645	6.605	5.785

From Tables 1 and 2, we can observe the better PSNR and bit error rate as compared to the other state of the art methods after embedding the watermark information in 3D mesh models.

### Distortion rate between the host and watermarked model

The robustness of the proposed watermarking method is analyzed for the distortions created due the embedded watermark. To evaluate the robustness of the proposed method, the distortion rate ‘*DR*’ was calculated as follows (Eq. 20):

20 $$DR=\frac{\#FN[D]}{D}$$

**Figure 9:**
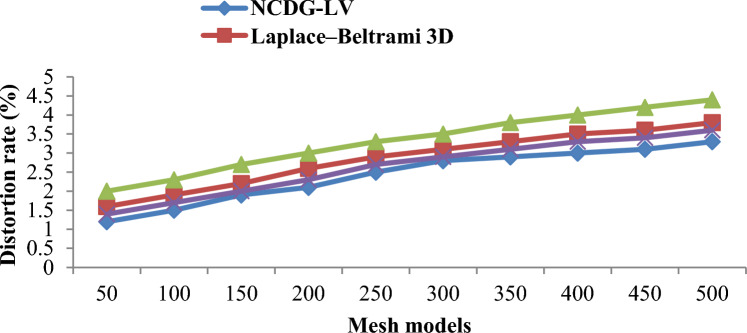
Graphical representation of distortion rate.

Distortion rate refers to the amount that a watermark is not detected in the attacked 3D mesh model and is based on the number of detected false negatives ‘$\#FN[D]$’ and the total number of detections ‘$D$’. In other words, a smaller distortion rate value is desirable. Herein, the Levenberg–Marquardt Deep Learning Extraction algorithm is evaluated on fifty distinct 3D mesh models, and the resultant results are shown in Fig. 9. Despite the very high number of 3D mesh models for the simulation, the proposed method achieved a smaller distortion rate compared to Laplace–Beltrami 3D^18^, 3D-MDAQIM^5^, and Deep 3D mesh watermarking network^23^.

Figure 9 presents the graphical representation of distortion rate using the three approaches for 50–500 different 3D mesh models. The 3D Mesh distortion rate refers to the degradation process involved in the watermarked 3D mesh model due to secret embedding. From the results, it can be inferred that the distortion rate obtained by the three different methods is directly proportional to the number of 3D mesh models provided as input. In other words, by increasing the number and size of 3D mesh models, a significant amount of distortion is also said to occur. As the rate of embedding increases, a small amount of visual degradation is caused, therefore resulting in the distortion. For 50 3D mesh models, the distortion rate of NCDG-LV, Laplace–Beltrami 3D^18^, 3D-MDAQIM^5^, and Deep 3D mesh watermarking network^23^ was found to be 1.2, 1.6, 2 and 1.4 respectively. The reason behind the improvement of the proposed method can be owed to the incorporation of multi-function barycenter using Levenberg-–Marquardt, which separates the top half and bottom half of the segmented sub-regions and subsequently reduces the distortion.

### Performance analysis of true positive rate

The true positive rate, or sensitivity, refers to the percentage of testing models that have been properly authenticated with a watermark. The true positive rate ‘$TPR$’ is measured using Eq. (21):

21 $$TPR= \frac{TP}{TP+FN}$$

**Figure 10:**
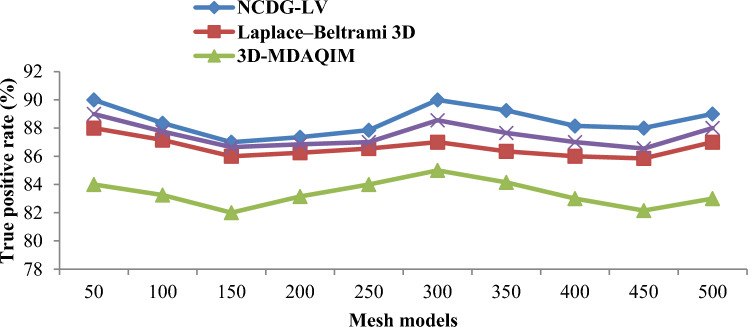
Graphical representation of true positive rate.

True positive rate is based on the true positives ‘$TP$’ (authenticated with the 3D mesh watermark) and false negatives ‘$FN$’ (not authenticated with 3D mesh watermark but is assumed to be authenticated). To demonstrate the watermark authentication efficiency of the proposed NCDG-LV, the obtained true positive rate was compared to those of three other methods, as seen in Fig. 10. Figure 10 presents the true positive rate results of the three methods in the watermark authentication process. As shown in the graphical results, the true positive rate of three different methods gets increased or decreased while increasing or decreasing the input from 50 and 500. This is because the true and false watermark authentication depends on the detection of salient points being detected and on the map segmentation performed for the detected points. The obtained true positive rate was compared to those of three other methods. From the results show that the true positive rate was found to be 90%, 88%, 84% and 89% using the four methods, respectively. The better performance is attained due to the enhancements generated by the multi-function barycenter and Levenberg–Marquardt Deep Learning Extraction functions.

### Watermark extraction against different attacks

To prove the robustness of the proposed approach, the watermarked model is tested with smoothing, Gaussian noise, cropping, and translation attacks on four different methods, including NCDG-LV, Laplace–Beltrami 3D^18^, and 3D-MDAQIM^5^, and Deep 3D mesh watermarking network^23^.

#### Smoothing attack

**Figure 11:**
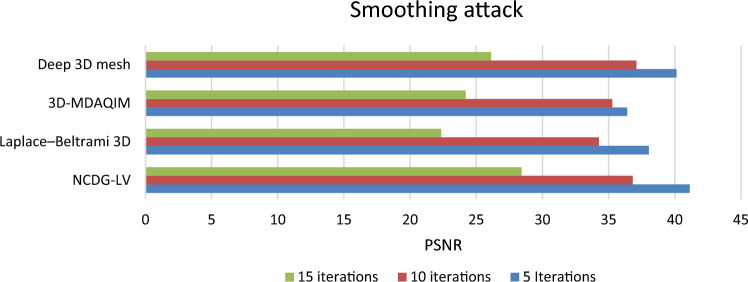
Average PSNR of extracted watermark after smoothing attack over 50 mesh models.

In more complex models, you might want to retain some degree of sharpness in certain areas while smoothing others. A surface smoothing algorithm is applied to test the robustness of the proposed method over 5, 10 and 15 iterations and compared with other state of the art methods in terms of PSNR and shown in Fig. 11.

The bit error rate of the proposed NCDG-LV and existing methods^7,18,22^ is calculated with smoothing attack (of 10 iterations) and without smoothing attack and is shown in Table 3. The bit error rate (training: 7.78%, testing: 5.64%) found to be significantly reduced using the proposed NCDG-LV method both with and without smoothing attack compared to Laplace–Beltrami 3D (8.79%, 5.65%)^18^, 3D-MDAQIM (9.65%, 6.61%)^5^, and Deep 3D mesh^23^ (8.34%, 5.78%).

**Table 3:** Bit error rate of three methods with smoothing attacks.

Model	NCDG-LV	Laplace–Beltrami 3D	3D-MDAQIM	Deep 3D mesh
Model1	3.25	3.85	4.15	3.55
Model2	3.85	4.25	4.55	4.05
Model3	3.55	4.55	4.65	3.75
Model4	4.35	4.85	5.35	4.65
Model5	5.35	6.15	7.25	5.85
Model6	7.25	8.35	9.00	7.85
Model7	8.55	9.00	10.35	8.75
Model8	10.35	11.55	12.00	11
Model9	15.00	17.35	19.15	16.55
Model10	16.35	18.00	20	17.45
Average	7.785	8.79	9.645	8.345

#### Gaussian noise attack

**Figure 12:**
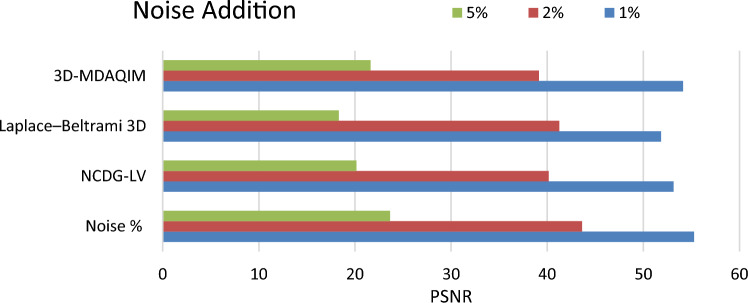
PSNR for the original watermark and extracted watermark with noise addition of 1%, 2%, and 5%.

Gaussian noise of 1%, 2%, and 5% is added to the models to test the robustness against noise attacks and the amount of distortion after adding noise is shown in Fig. 12 as a value of PSNR and the same is compared against the other state of the methods and shown in Table 4.

**Table 4:** PSNR of NCDG-LV method, Laplace–Beltrami 3D ^18^, and 3D-MDAQIM ^5^, Deep 3D mesh ^23^ on a set of 10 distinct 3D mesh models with 1%, 2%, and 5% noise.

Model	NCDG-LV	Laplace–Beltrami 3D	3D-MDAQIM	Deep 3D mesh
Model1	51.05	49.12	46.19	50.38
Model2	53.55	51.05	47.53	52.46
Model3	47.53	41.51	40.17	45.58
Model4	53.55	49.13	46.19	51.45
Model5	49.12	47.53	45.03	48.55
Model6	51.05	49.12	46.19	49.68
Model7	46.19	44.01	41.27	45.52
Model8	51.05	49.13	47.53	50.22
Model9	47.53	46.19	45.03	46.88
Model10	51.05	49.13	47.53	49.95

#### Cropping attack

**Figure 13:**
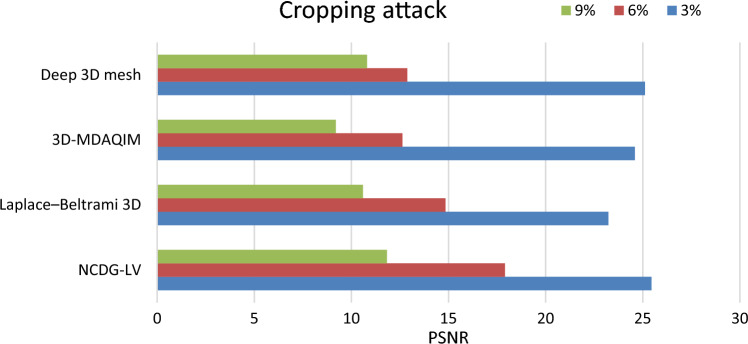
PSNR for the original watermark and extracted watermark after cropping the model for 3%, 6%, and 9%.

The 3D mesh models are cropped 3%, 6%, and 9% to test the robustness against cropping attacks and the amount of distortion after cropping is shown in Fig. 13 as a value of PSNR.

The experimental results show that the proposed method exhibits good robustness against attacks mentioned above, and the segmented watermarking provides better visual quality on an image compared with Laplace–Beltrami 3D^18^, 3D-MDAQIM^5^, and Deep 3D mesh watermarking network^23^. The Nearest Centroid Discrete Gaussian and Levenberg–Marquardt method displays high imperceptibility and robustness. The watermarked images were corrupted by Gaussian noise of 1% variance. For the cropping attack, a small portion of the watermarked image was removed.

### Ablation analysis

Ablation analysis was carried out to analyze the impact of different modules of the proposed NCDG-LV technique for enhanced watermark embedding and extraction of 3D model is shown in Figs. 10, 11, 12, and 13. It is evident that the model’s overall performance gradually improves with the inclusion of the various process. The metrics employed to demonstrate the performance of the proposed NCDG-LV 3D model watermarking are PSNR, bit error rate, distortion rate and TPR. The performance of the proposed method is evaluated based on various attacks like smoothing attack, Gaussian noise attack and cropping attack.

NCDG-LV model includes the following process: Salient point detection using Nearest Centroid and Discrete Gaussian geometric (NC–DGG) is considered as the baseline of the proposed model. This selected point regions are further segmented using Map Segmentation (MS). This region is embedded with secret watermark using multi-function Barycenter to generate the watermarked 3D model (MF_B). The watermarks are extracted using the Levenberg–Marquardt deep neural network (LM_DN). The ablation study is performed with other methods like selection of random vertices instead of salient points, clustering in the process of map segmentation, LSB embedding^26^ instead of barycentric method to show the improvements attained with respect to PSNR and Bit Error rate.

**Table:** 

Proposed modules	PSNR	
	Train set	Test set
Vertices + clustering + MF_B + LM_DN	22.68	25.84
NC_DGG + Fuzzy clustering + LSB + LM_DN	42.45	40.12
NC_DGG + MS + LSB + LM_DN	52.25	48.12
Proposed NCDG-LV (NC–DGG + MS + MF_B + LM_DN)	58.12	54.13

## Conclusion

An effective Nearest Centroid Discrete Gaussian and Levenberg–Marquardt watermarking method for 3D mesh authentication is presented. In this technique, a novel geometric Nearest Centroid Discrete Gaussian is used to identify the salient points, and bits are embedded into the segmented portion of a 3D mesh using multi-function barycenter embedding. This, in turn, helps to reduce the distortion rate. In addition, Levenberg–Marquardt extraction is applied to extract the watermarked image. The experimental results demonstrate the proposed method achieves good imperceptibility and robustness against attacks and is comparatively better to other state-of-the-art methods in terms of salient point detection time, distortion rate, and true positive rate.

In future we would like to explore watermarking techniques that dynamically adapt the embedding strength of the watermark in accordance with the sensitivity of the mesh model. And, explore new strategies for watermark placement within 3D mesh models that are intelligently coordinated with the inherent semantic features of the content to provide effective protection with the least amount distortion.

## Data Availability

The datasets analyzed during the current study are available in the Princeton Shape Benchmark dataset repository, https://shape.cs.princeton.edu/benchmark/classifications/v1.
